# Healthy and Sustainable Diet Index: Development, Application and Evaluation Using Image-Based Food Records

**DOI:** 10.3390/nu14183838

**Published:** 2022-09-16

**Authors:** Amelia J. Harray, Carol J. Boushey, Christina M. Pollard, Satvinder S. Dhaliwal, Syed Aqif Mukhtar, Edward J. Delp, Deborah A. Kerr

**Affiliations:** 1 Curtin School of Population Health, Curtin University, Bentley, Perth, WA 6102, Australia; 2 Children’s Diabetes Centre, Telethon Kids Institute, Nedlands, Perth, WA 6009, Australia; 3 Epidemiology Program, University of Hawaii Cancer Centre, Honolulu, HI 96813, USA; 4 Department of Nutrition, Purdue University, West Lafayette, IN 47907, USA; 5 Curtin Health Innovation Research Institute, Curtin University, Kent Street, GPO Box U1987, Perth, WA 6845, Australia; 6 Duke-NUS Medical School, National University of Singapore, 8 College Rd, Singapore 169857, Singapore; 7 Institute for Research in Molecular Medicine (INFORMM), University Sains Malaysia, Minden 11800, Penang, Malaysia; 8 Office of the Provost, Singapore University of Social Sciences, 463 Clementi Road, Singapore 599494, Singapore; 9 School of Electrical and Computer Engineering, Purdue University, West Lafayette, IN 47907, USA

**Keywords:** healthy and sustainable diet index, mobile food record, images, image-based dietary assessment, environmental sustainability, young adults

## Abstract

There are limited methods to assess how dietary patterns adhere to a healthy and sustainable diet. The aim of this study was to develop a theoretically derived Healthy and Sustainable Diet Index (HSDI). The HSDI uses 12 components within five categories related to environmental sustainability: animal-based foods, seasonal fruits and vegetables, ultra-processed energy-dense nutrient-poor foods, packaged foods and food waste. A maximum of 90 points indicates the highest adherence. The HSDI was applied to 4-day mobile food records (mFR^TM^) from 247 adults (18–30 years). The mean HSDI score was 42.7 (SD 9.3). Participants who ate meat were less likely to eat vegetables (*p* < 0.001) and those who ate non-animal protein foods were more likely to eat more fruit (*p* < 0.001), vegetables (*p* < 0.05), and milk, yoghurt and cheese (*p* < 0.05). After adjusting for age, sex and body mass index, multivariable regression found the strongest predictor of the likelihood of being in the lowest total HSDI score tertile were people who only took a bit of notice [OR (95%CI) 5.276 (1.775, 15.681) *p* < 0.005] or did not pay much/any attention to the health aspects of their diet [OR (95%CI) 8.308 (2.572, 26.836) *p* < 0.0001]. HSDI provides a new reference standard to assess adherence to a healthy and sustainable diet.

## 1. Background

As evidence linking dietary behaviours to climate change is advancing, national dietary guidelines are incorporating specific advice to inform the public and guide policy [1]. People can lower the environmental impact of their dietary behaviours without sacrificing nutrient intake by adopting a more sustainable diet, and in many scenarios, can improve health and reduce risk of premature mortality [2]. By adopting more environmentally sustainable dietary behaviours, such as shifting to more plant-based proteins, people can support sustainable food systems and reduce the impact of dietary patterns on the climate [3]. The EAT-Lancet Commission on the environmental sustainability of food and the impacts of food consumption on health recommended that to achieve sustainable food systems, a shift to healthy and sustainable dietary patterns is needed [3]. Despite increasing recognition of the need for sustainable, resilient food systems for healthy diets and the acknowledgement of the need for a universal ‘healthy reference diet’, many challenges remain [4]. The commission’s ‘planetary health plate’ recommendations are designed to be flexible, acknowledging that a reference diet would need to accommodate cultural differences, traditional eating patterns and individual preferences. A carefully selected diet that meets environmental needs can meet all nutrient requirements [1].

Sustainable diets are defined by the Food and Agriculture Organization as “those diets with low environmental impacts which contribute to food and nutrition security and to healthy life for present and future generations” [5]. In Australia, healthy diet recommendations conform to the Australian Dietary Guidelines (ADGs) [6]. The ADGs provide food-based recommendations and direction for nutrition policy, yet they contain no specific guidelines on sustainable dietary behaviours. This is despite reporting on a strengthening evidence base and recognition of its importance since 2003 [3,7]. Several individual guidelines support a diet for good health and also support a diet to reduce the burden on the environment and food system [8], but this has not been purposively examined.

There is no agreed definition for what constitutes a healthy and sustainable diet, making it challenging for ongoing population monitoring and for assessing the impact of diet on health outcomes. An additional challenge is the limited evidence on how current dietary behaviours align with the multidimensional nature of sustainable diets. Filling this gap could inform government policies that support two major public health issues: poor health and climate change [3,9,10,11]. As the evidence of the food supply’s impact on the environment strengthens, there is an opportunity to develop a culturally acceptable and context-specific diet quality index (DQI) to accurately assess sustainable diets [12].

Commonly used dietary assessment methods, such as a 24 h dietary recall (24 HR) and Food Frequency Questionnaires (FFQ), have been used to assess adherence to the ADGs [13]. One or a combination of these methods can be used to measure behaviours that align with a sustainable diet, and have been done so by Stubbendorff and colleagues [14]. However, due to the reliance on memory and retrospective portion size estimation, they may less accurately measure the multidimensional nature of healthy and sustainable (H&S) diets. For example, using these methods to assess individual food packaging, seasonal produce and plate waste would further increase reliance on memory and on the time required to complete, potentially impacting the accuracy of the data collected. The mobile food record (mFR^TM^) is an image-based mobile application [15,16,17] with the potential to capture additional sustainable eating behaviours, such as edible plate waste, individually packaged foods and seasonality, without placing additional burden on respondents or relying on their recall ability or literacy levels [8].

Diet quality indices (DQI) reflect dietary patterns, adherence to dietary guidelines in populations over time, and association of dietary intakes with health outcomes, as well as inform nutrition messages, research, and policy [18]. One strength of DQIs is that they consider the multidimensional nature of diets and apply weighting factors to each component to calculate a final adherence score. Internationally, groups have developed sustainable diet indices using dietary intake data collected via FFQ [19,20] and repeated 24 HR recall [21]. However, to the authors’ knowledge, no diet quality index has been developed to measure compliance with an H&S diet using image-based mFR^TM^, and such a tool could provide additional contextual information to help inform policy makers [12].

The aim of this study was to develop a theoretically derived Healthy and Sustainable Diet Index (HSDI) to determine a density score, and then apply the index on dietary intake using images captured using the mFR^TM^.

## 2. Methods

### 2.1. Study Sample

The population-based sample consisted of 247 adults aged 18 to 30 years who were recruited via the Federal Electoral Roll during the Connecting Health and Technology (CHAT) study [22]. Data were collected from the same participants on two separate occasions, six months apart. Further details about the methods have been previously outlined by the authors [22]. The CHAT study was registered on the Australian and New Zealand Clinical Trials Registry (ACTRN12612000250831) and approved by the Curtin University Human Resources Ethics Committee (HR181/2011) and the Western Australian Department of Human Research Ethics Committee (#2011/90).

### 2.2. Assessment of Healthy and Sustainable Dietary Behaviours

**Figure 1:**
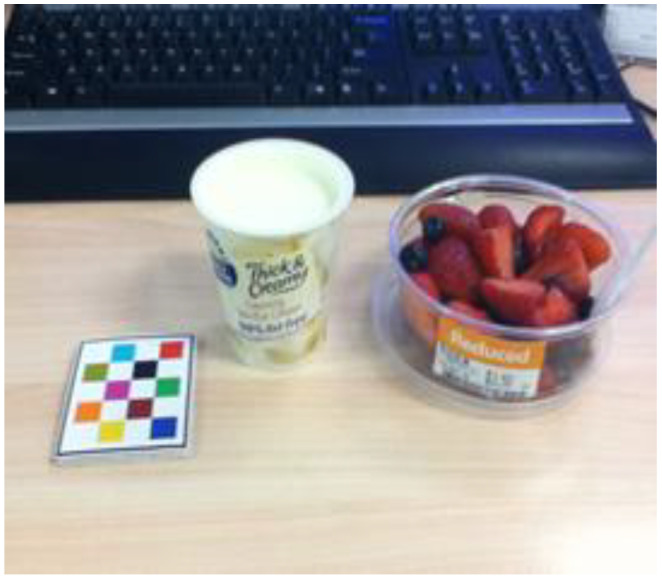
Example of assessment of individually packaged foods using the mobile food record™.

The protocol outlining the justification and the methods used to assess individual H&S dietary behaviours have previously been published by the authors [8]. Briefly, 4-day image-based mFR^TM^ were used, in which participants captured images of all the foods and beverages they consumed over four consecutive days. The H&S dietary behaviours were assessed by a trained analyst using images captured with the mFR^TM^, including (1) intake of animal-based foods, including ruminant meat, pigs, poultry, fish, seafood, dairy foods and eggs; (2) intake and seasonality of fruits and vegetables (including legumes and beans) and other plant-based foods high in protein (including nuts, seeds and tofu); (3) intake of ultra-processed energy-dense nutrient-poor (EDNP) foods and beverages, as defined by Monteiro and colleagues [23], including sugar-sweetened beverages (SSB) and alcohol; (4) use of individually packaged foods (see Figure 1); and (5) food (plate) waste behaviours.

The theoretically driven HSDI contains twelve individual items related to H&S dietary behaviours. The items chosen to be included in any diet quality index are a compromise between what information is available and what information is practical to include, which is often driven by the dietary assessment method used to collect the data. Therefore, deciding on the dietary components included and excluded involves an element of researcher subjectivity. Details explaining greater detail of the components included in the HSDI have been described by the authors, previously [8]. The individual items and their respective weightings can be seen in Table 1. The influence of dietary behaviours on human health were given the highest weighting, followed by evidenced impact on the environment. This was due to stronger evidence in this area of dietary intake and health outcomes. A maximum score of either five or ten points was allocated to each component of the HSDI. To determine the weighting, each component in the HSDI was categorised into one or more of the following elements: impact on human health and/or impact on the environment. For example, ultra-processed EDNP foods and beverages affect health (contributing excess kilojoules (kJ) and contributing to chronic disease risk) and the environment through the use of land, water, electricity, transport, packaging, storage and disposal [24]. Therefore, these foods and beverages were given a maximum weighting of ten points each. Another example is food waste, which has a direct negative impact on the environment (resources used to dispose of waste and landfill) and a potential influence on health, as fresh fruit and vegetables are perishable and often thrown away, creating a barrier for purchase and consumption. However, due to limited evidence for the latter, food waste was assigned a maximum of five points (for an average of ≤10% of edible plate waste over the 4-day mFR). Ten points were allocated to behaviours that positively or negatively align with both a healthy *and* sustainable diet (such as vegetables), and a maximum of five points were allocated to other dietary behaviours, such as food packaging.

The HSDI used a continuous weighting system based on increments of food group serves, or proportions of total intake for behaviours without set recommendations, such as seasonality of fruits and vegetables and plate waste. In a review of diet quality indices, the method of using continuous scales (opposed to simple cut offs or binary scales) has been identified as a superior method because the intake of many foods has a “U-shaped” effect [25], and continuous scales allow for more variability and provide more sensitivity within the index. For the present study, determining the categories for the continuous scale involved developing a maximum score for each component (as described above), then dividing it into five categories, each with different scores assigned. In circumstances where these dietary behaviours were reflected in the ADGs (such as fruit and vegetables, and milk, yoghurt and cheese) the maximum weighting assigned to the item was determined by the ADGs recommended daily number of serves [6]. In circumstances where the behaviour was not reflected in the ADGs, such as seasonality, food waste or individual food packaging, categories were created from the scientific literature.

Some food groups in the ADGs were separated into several items in the HSDI due to notable differences in the environmental impact of these foods. For example, the “lean meat, poultry, fish and alternatives“ food group was separated into “ruminant meat and pig”, “poultry and fish“ and “non-animal protein alternatives”. To minimise foods being picked up in two or more components of the index, for example, legumes and beans appear in both the “vegetable” and “lean meat and alternatives” food group, were assigned to the “non-animal protein foods” group. Some foods, such as EDNP foods may have been counted in both the categories of “individually packaged foods” and “ultra-processed EDNP foods”. However, to minimise repetition, foods such as ice cream were only counted as ultra-processed EDNP foods, and not “milk, yoghurt and cheese”.

### 2.3. Statistical Analyses

A purpose-built Microsoft Access Database was used to record the components of an H&S diet. This study involved a secondary analysis of 4-day mFR^TM^ collected from a population-based sample at baseline (*n* = 247) and six months (*n* = 220). Once the secondary analysis of all mFR^TM^ (approximately 12,000 food images) was complete, the dietary data was exported to SPSS Version 22 and merged with the participants’ anthropometric and demographic characteristics. SPSS Version 22 was used for all data analyses and *p*-values of less than 0.05 were considered statistically significant.

Five stages of analysis were conducted to assess the following:

1. Descriptive statistics about the sample, including demographic, anthropometric, and dietary variables.
2. The specific dietary differences between participants with the lowest, middle, and highest total HSDI scores. This was conducted by separating the participants’ total HSDI scores into tertiles using the SPSS rank function. One-way ANOVA was used for continuous variables (age and body mass index (BMI)) and the Chi-Squared test for all remaining categorical variables.
3. The relationship between the components of the index to assess if H&S dietary behaviours are related. This was conducted using the non-parametric test, Spearman’s correlation coefficient.
4. Regression analyses were conducted to assess which variables help determine the characteristics of those who are in the lowest tertile for total HSDI score and whether any individual variables were predictors of overall HSDI score. Univariate regression analyses were conducted to identify which individual variables predict those most at risk of being in the lowest tertile of HSDI score (20–38 out of 90). Univariate regression analyses were then conducted after adjusting for age, sex and BMI. Multivariate regression analyses were conducted to see which variables continued to determine those most at risk of being in the lowest tertile when including all variables in the model.
5. The test–re-test reliability of the index was assessed by comparing individual components and the overall HSDI score of participants who completed the CHAT study (mFR^TM^ collected at baseline and at the six-month visit (*n* = 220)).

## 3. Results

Descriptive statistics from the 247 participants are outlined in Table 2. One participant was excluded from the analysis due to an incomplete food record. 77.2% of the participants were White, 58.5% had a BMI in the healthy weight range (18.5–24.9 kg/m^2^), 32.1% were classified as either overweight or obese, and 37.4% reported taking vitamin supplements. The mean intake of fruit was 0.9 (±0.7) serves per day and the mean intake of vegetables was 1.8 (±1.0) serves per day. More than half (51.4%) of the fruits and vegetables consumed were in season in Western Australia at the time of consumption, and 20% of edible food prepared and served was assessed as edible plate waste.

The intake of individual components of the HSDI are shown in Table 2. The mean intake of ultra-processed EDNP foods and sugar sweetened beverages (one serve is equivalent to about 600 kJ) over 4 days was 2.7 (±1.4) and 1.0 (±1.0) serves per day, respectively, and participants consumed a mean of 1.9 (±1.4) individually packaged EDNP items (such as a chocolate bar or can of SSB) and 1.6 (±1.2) individually packaged healthy items (such as a bottle of water or small tub of yoghurt) per day.

People who reported taking vitamin supplements in the self-reported written questionnaire were significantly more likely to have a higher HSDI score than those who did not (*p* < 0.005). Those who reported paying a lot of attention to the health aspects of their diet were more likely to have a higher total HSDI score than those who reported not thinking much or at all about the health aspects of the food they eat (*p* < 0.0005). There were statistically significantly differences in participants in each tertile and scores for individual components of the index (Table 3), which is expected, as the tertiles were ranked on total HSDI score, taking into account all components. However, the intake of seasonal fruits and vegetables; ruminant animal meat and pigs; and milk, yoghurt and cheese were exceptions to this. There were no significant differences detected between the tertiles. See Table 3 for the differences between the HSDI scores for each tertile.

Spearman’s correlation test results indicate participants who ate ruminant meat and pigs were significantly less likely to eat vegetables (*p* < 0.001) (Table 4). Those who consumed milk, yoghurt and cheese were significantly more likely to eat vegetables (*p* < 0.05). In addition, those who ate non-animal protein foods, such as legumes, tofu, nuts and seeds were significantly more likely to eat more fruit (*p* < 0.001), vegetables (*p* < 0.05) and dairy foods (*p* < 0.05). The strongest association found was between the intake of individually packaged EDNP foods and ultra-processed EDNP foods (*p* < 0.001) and EDNP beverages (*p* < 0.001) (Table 4).

The univariate analyses showed that those not taking vitamin supplements were more likely to have an HSDI score in the lowest tertile (OR = 1.855, 95%CI [1.059, 3.250], *p* < 0.05) (Table 5). This relationship was still significant after adjusting for age, sex and BMI, however, it was ruled out as a predictor for being in the lowest tertile of HSDI scores once all variables were taken into account in the multivariate regression. Participants who reported currently smoking were significantly more likely to be in the lowest tertile (OR = 3.407, 95%CI [1.065, 10.904], *p* < 0.05). However, after adjusting for age, sex and BMI, no significant association was observed. The strongest predictor of the likelihood of being in the lowest tertile for total HSDI score was dietary health consciousness. After adjusting for all other variables in the multivariate regression model, those who reported only taking a bit of notice (OR = 5.276, 95%CI [1.765, 15.619], *p* < 0.005) or not thinking much or at all about the health aspects of their diet (OR = 8.308, 95%CI [2.572, 26.836], *p <* 0.0001) were more likely to be in the lowest tertile of HSDI scores. Table 5 shows results from the univariate and multivariate regression analyses.

The test–re-test reliability of the HSDI was assessed using data collected from the same sample on two different occasions, six months apart (*n* = 220) (Table 6). The results indicated significant differences between the baseline and six-month visit for all components of the index, with the exception of non-animal protein foods and poultry, fish and eggs. The difference between the total HSDI scores for participants from baseline to the six-month visit was 4.1 points (*p* < 0.0005), with the six-month visit having improved HSDI scores (closer adherence to a healthy and sustainable diet).

## 4. Discussion

The aim of this study was to develop a theoretically derived Healthy and Sustainable Diet Index (HSDI) to determine a density score, and then apply the index on dietary intake using images captured by the mobile food record (mFR™). The findings suggest people who reported taking vitamin supplements had an increased likelihood of having diets more aligned with an H&S diet, regardless of age, sex or BMI. Previous studies have found those who are least at risk of poor nutrient intake are more likely to use nutritional supplements [27]. However, no research has examined the relationship between sustainable dietary behaviours and supplement use. Another key finding was that dietary health consciousness was the only independent predictor of one’s likelihood of being in the lowest tertile of HSDI scores, when all other variables were included in the multivariate regression model. These findings support the inclusion of a measure of dietary health consciousness (e.g., “Which of the following best describes how you feel about the health aspects of your diet?*”)* in future sustainable diet research. This question has been associated with high level of concern regarding the impact of the environment on the food supply [28] and EDNP food, SSB intake [29] and support for government interventions to promote dietary guidelines [30].

The evaluation of the HSDI found that individual components were related to each other, with people who displayed one behaviour being significantly more or less likely to also display another behaviour. For example, the intake of non-animal protein foods was associated with fruit (*p <* 0.001) and vegetable intake (*p <* 0.001), and all were aligned with an H&S diet. Conversely, the intake of EDNP foods, beverages and individually packaged foods were associated with one another, and all were unsupportive of a sustainable diet. The ability to detect these associations shows an element of sensitivity within the index. Further research applying principle component analysis to the HSDI scores will help determine if there are independent components of the Index and if any components can be ruled out.

To date, there is no evidence available on the use of individually packaged foods by consumers in Australia. The novel findings in this study show these foods and beverages, both EDNP and healthy individually packaged items, are consumed daily (1.9 ± 1.4 items and 1.6 ± 1.2 items per day, respectively). This dietary behaviour is of concern considering a sustainable diet, as ultra-processed EDNP foods and beverages: (1) provide minimal, if any, nutritional value; (2) encourage the overconsumption of kilojoules above energy requirements; and (3) require resources (such as water and electricity) for the extensive levels of food processing, and (4) food packaging. On a positive note, these findings confirm the value of recommending a dietary pattern for individual health and sustainability. Conducting image-based dietary assessment using the mFR^TM^ to measure the use of individually packaged foods and edible food waste in real time was a unique aspect of this study.

The low mean HSDI scores for fruits (4.6 ± 3.3 out of a possible points) and vegetables (3.9 ± 2.1 out of a possible points) were unsurprising, as national population dietary surveys have shown poor compliance with recommendations for these food groups [31]. The high intake of EDNP foods were similarly expected, and the mean scores (2.1 ± 2.4 out of a possible 10 points) reflect the estimated 36% of the total daily energy intake of Australian adults coming from EDNP foods [31].

The study sample was predominantly female in the healthy weight range, although the mean BMI for both males and females was at the upper end with 24.7 and 24.1 kg/m^2^, respectively. The strengths of this study were that a population-based sample of participants was recruited via the Federal Electoral Roll and data were collected from the same participants on two separate occasions, six months apart. This enabled the test–re-test reliability of the index to be evaluated using a paired sample *t*-test.

The HSDI is the first diet quality index to use image-based food records to assess dietary behaviours that influence health outcomes (e.g., EDNP foods and beverages) and those that significantly burden the environment (e.g., individually packaged foods and food waste), which are often not assessed using traditional dietary assessment methods. In the absence of a gold standard for an H&S diet and the rudimentary evidence on the environmental impact of specific food groups in an Australian context, challenges arose when it came to its evaluation. Although validation of all dietary assessment methods is important to measure whether they accurately achieve their goals, first, a ‘gold standard’ is required [32]. Such a standard requires a strong evidence base, such as the Dietary Guidelines for good health, and does not exist regarding an H&S diet in Australia. First, a reference standard is required [32]. The present study developed a new reference standard to examine H&S diets using images, which can be used in future studies and applied to a larger population group and wider age range. The HSDI maximum score of 90 points was developed as a result of equal weighting of the elements of a healthy and/or sustainable diet that could be retrospectively assessed using the mFR^TM^ images. There is potential to modify the weighting of individual components of the HSDI, and include additional components, as evidence on the environmental impact of foods evolves, and if the HSDI is applied to different settings.

The HSDI demonstrated its ability to assess the multidimensional nature of an H&S diet by incorporating 12 components into the index and finding significant associations between behaviours. Future research involving the application of the HSDI to a larger, more diverse sample, the collection of markers of health outcomes (such as blood lipid profiles as a risk factor for cardiovascular disease), and additional dietary behaviours, such as the use of nutritional supplements, will strengthen the evaluation of the index.

Similar to other methods, using the mFR^TM^ to assess diet is accompanied by limitations. The primary limitation being participants forgetting to capture an image of an eating occasion. This can be minimised by the ability to set alerts on the mobile device to remind participants to capture images of all foods and beverages consumed. These alerts have previously been described by Ahmad and colleagues [33]. Another limitation is the potential for estimation error inherent in dietary assessment methods involving humans, including the use of a trained analyst in this study. The advancements in the use of mFR^TM^ technology toward automated image analysis in the future may reduce this error. The influence of social desirability bias is a potential limitation of this study. In addition, due to the secondary analysis of existing data, the dietary behaviours of focus were limited to those collected from existing mobile food records during the CHAT study. As this study involved a secondary analysis of all mFR^TM^ collected at baseline and at the six-month return visit of the CHAT study, the authors were limited by the data collected in this study. The future directions of this research could amend the mFR^TM^ to collect additional information from participants, such as the assessment of nutritional supplement use through images and prompts to ask whether their food waste and packaging was put into landfill, recycling or composted.

Further research exploring consumer interest and awareness of H&S diets and modifications to the existing mFR^TM^ app would strengthen the proposed method. For example, short survey questions could be included in the app to measure variables, such as dietary health consciousness or supplement use, to increase the level of detail collected for the dietary assessment method.

## 5. Conclusions

Dietary guidelines to encourage and promote behaviours that support good health and environmental sustainability are needed to inform and guide nutrition policy. Evidence on how current dietary patterns align with healthy and sustainable diets will highlight the need for these guidelines in Australia. This study provides a new reference standard for the Healthy and Sustainable Diet Index. Using the image-based mobile food record^TM^, dietary behaviours known to have a greater impact on the environment can be assessed without placing additional burden on users. The novel index using the mFR^TM^ is a prediction model that can be applied to other population groups and datasets to further evaluate its ability to measure adherence to a healthy and sustainable diet.

## Figures and Tables

**Table 1:** Components of the Healthy and Sustainable Diet Index, in ascending order of alignment with a H&S diet (maximum score of 90) *.

Item	Item Description	Lowest HSDI Score				Highest HSDI Score	Maximum Item Score
1	Fruit ^a^	0 serves (*0 points*)	0.01–0.5 serves (*2 points*)	0.51–1.25 serves (*5 points*)	1.26–1.99 serves (*8 points*)	≥2 serves (*10 points*)	10
2	Vegetables ^a^	<0.5 serve (*1 point*)	0.5–1.5 serves (*2 points*)	1.51–3 serves (*5 points*)	3.01–4.99 serves (*8 points*)	≥5 serves (*10 points*)	10
3	Seasonality of fruits and vegetables ^b^	0–20% (*1 point*)	20.1–40% (*2 points*)	40.1–60% (*3 points*)	60.1–80% (*4 points*)	>80% (*5 points*)	5
4	Ruminant animal meat and pigs ^a^	>3 serves (*0 points*)	2.01–3 serves (*1 points*)	1.01–2 serves (*2 points*)	< 0.25 serve (*4 point*)	0.25–1 serve (*5 points*)	5
5	Poultry, fish and eggs ^a^	>3 serves (*0 points*)	2.01–3 serves (*2 points*)	< 0.25 serve (*3 point*)	1.01–2 serves (*4 points*)	0.25–1 serve (*5 points*)	5
6	Milk, yoghurt and cheese ^a^	<0.5 serve (*1 points*)	0.5–1 serve (*2 points*)	1.01–2 serves (*3 points*)	>2.5 serves (*4 points*)	2.01–2.5 serves (*5 points*)	5
7	Non-animal protein foods (legumes, tofu, nuts, seeds) ^a^	0 serves (*0 points*)	0.01–0.75 serves (*2 points*)	0.76–1.75 serves (*6 points*)	1.76–2.5 serves (*8 points*)	>2.5 serves (*10 points*)	10
8	EDNP foods ^a^	>2.75 serves (*0 points*)	1.76–2.75 serves (*2 points*)	0.76–1.75 serves (*4 points*)	0.01–0.75 serves (*8 points*)	0 serves (*10 points*)	10
9	Unhealthy beverages (SSBs and alcohol) ^a^	>2 serves (*0 points*)	1.26–2 serves (*2 points*)	0.51–1.25 serves (*4 points*)	0.01–0.50 serves (*8 points*)	0 serves (*10 points*)	10
10	Individually packaged EDNP foods and beverages	>2.25 items (*0 points*)	1.51–2.25 items (*2 points*)	0.76–1.5 items (*3 points*)	0.01–0.75 items (*4 points*)	0 items (*5 points*)	5
11	Individually packaged healthy foods and beverages	>2.25 items (*2 points*)	1.51–2.25 items (*4 points*)	0.76–1.5 items (*6 points*)	0.01–0.75 items (*8 points*)	0 items (*10 points*)	10
12	Edible plate waste	>40% (*1 point*)	30.1–40% (*2 points*)	20.1–30% (*3 points*)	10.1–20% (*4 points*)	≤10% (*5 points*)	5
Total maximum score for each category		6 points	25 points	47 points	72 points	90 points	

* A higher HSDI score indicates closer alignment to a healthy and sustainable diet; ^a^ Serve sizes according to the Australian Guide to Healthy Eating [6]; ^b^ Percentage of fruits and vegetables in season at time of consumption, calculated from automated time and date stamp against WA seasonality chart.

**Table 2:** Descriptive statistics of the study population and HSDI index scores * at baseline (*n* = 246).

Variable	Description	Men (*n* = 85)	Women (*n* = 161)	Total (*n* = 246)
		Total score	Total score	Total score
Age	Years	24.6 ± 3.3	24.2 ± 3.4	24.3 ± 3.4
Body Mass Index	kg/m^2^	24.7 ± 4.4	24.1 ± 5.8	24.3 ± 5.3
		n (%)	n (%)	n (%)
Ethnicity	White	68 (80.0)	122 (75.8)	190 (77.2)
	Asian	9 (10.6)	32 (19.9)	41 (16.7)
	Other	8 (9.4)	7 (4.3)	15 (6.1)
Body Mass Index	Underweight (<18.5 kg/m^2^)	7 (8.2)	16 (9.9)	23 (9.3)
	Healthy weight (18.5–24.9 kg/m^2^)	43 (50.6)	101 (62.7)	144 (58.5)
	Overweight (25–29.9 kg/m^2^)	37 (31.8)	22 (13.7)	49 (19.9)
	Obese (≥30 kg/m^2^)	8 (9.4)	22 (13.7)	30 (12.2)
Body Mass Index	Healthy weight and below (<25 kg/m^2^)	50 (58.8)	117 (72.7)	167 (67.9)
	Overweight (25–29.9 kg/m^2^)	27 (31.8)	22 (13.7)	49 (19.9)
	Obese (≥30 kg/m^2^)	8 (9.4)	22 (13.7)	30 (12.2)
Vitamin supplement use	Yes	25 (29.4)	67 (41.6)	92 (37.4)
	No	60 (70.6)	94 (58.4)	154 (62.6)
Smoking status	Never smoked	53 (62.4)	116 (72.0)	169 (68.7)
	Previous smoker	25 (29.4)	39 (24.2)	64 (26.0)
	Current smoker	7 (8.2)	6 (3.7)	13 (5.3)
IPAQ category ^a^	Low activity (<600 MET mins/week)	7 (8.6)	25 (16.8)	32 (13.9)
	Moderate activity (minimum 600 MET mins/week)	39 (48.1)	86 (57.7)	125 (54.3)
	High activity (>3000 MET mins/week)	35 (43.2)	38 (25.5)	73 (31.7)
Education	Year 10, 11 or 12	32 (37.6)	56 (34.8)	88 (35.8)
	Trade or diploma	29 (34.1)	31 (19.3)	60 (24.4)
	University degree or higher	24 (28.2)	74 (46)	98 (39.8)
SEIFA ^b^	1–2	5 (5.9)	2 (1.2)	7 (2.8)
	3–4	2 (2.4)	12 (7.5)	14 (5.7)
	5–6	22 (25.9)	38 (23.6)	60 (24.4)
	7–8	9 (10.6)	41 (25.5)	50 (20.3)
	9–10	47 (55.3)	68 (42.2)	115 (46.7)
Dietary health consciousness ^c^	Pay a lot of attention to the health aspects of food	11 (12.9)	29 (18)	40 (16.3)
	Take a bit of notice to the health aspects of food	50 (58.8)	97 (60.2)	147 (59.8)
	Don’t think much or don’t think at all	24 (28.2)	33 (20.5)	57 (23.2)
Individual HSDI item scores		Mean ± SD	Mean ± SD	Mean ± SD
HSDI items with score 0–10 points	Fruit	4.4 ± 3.6	4.7 ± 3.1	4.6 ± 3.3
	Vegetables	4.0 ± 2.2	3.9 ± 2.1	3.9 ± 2.1
	Non-animal protein foods (legumes, nuts, seeds, tofu)	1.6 ± 1.9	1.8 ± 2.1	1.7 ± 2.0
	Ultra-processed EDNP foods	2.2 ± 2.7	2.0 ± 2.3	2.1 ± 2.4
	Ultra-processed beverages (SSBs and alcohol)	4.8 ± 3.6	5.4 ± 3.5	5.2 ± 3.6
	Individually packaged healthy foods and beverages	5.5 ± 2.8	5.3 ± 2.7	5.4 ± 2.7
HSDI items with score 0–5 points	Seasonal fruits and vegetables	3.1 ± 1.0	3.0 ± 1.1	3.0 ± 1.0
	Ruminant animal meat and pigs	3.4 ± 1.7	3.9 ± 1.5	3.7 ± 1.6
	Poultry, fish, eggs	4.0 ± 1.2	4.1 ± 1.1	4.1 ± 1.1
	Milk, yoghurt and cheese)	3.2 ± 1.1	2.9 ± 1.1	3.0 ± 1.1
	Individually packaged EDNP foods and beverages	2.2 ± 1.9	2.1 ± 1.7	2.1 ± 1.8
	Food (plate) waste	4.3 ± 1.2	3.6 ± 1.3	3.9 ± 1.3
Overall HSDI score	Out of 90 points	42.7 ± 9.7	42.7 ± 9.3	42.7 ± 9.3
HSDI items presented as serves per day ^d^, number of items or % of total	Fruit (serves/day)	1.1 ± 1.3	0.9 ± 0.7	0.9 ± 0.7
	Vegetables (serves/day)	1.8 ± 1.0	1.8 ± 1.0	1.8 ± 1.0
	Seasonal fruits and vegetables (% of total fruits and vegetables)	52.9 ± 20.4	51.4 ± 20.2	51.4 ± 20.2
	Ruminant animal meat (serves/day)	1.2 ± 0.9	0.8 ± 0.7	0.8 ± 0.7
	Poultry, fish, eggs (serves/day)	1.1 ± 0.8	1.0 ± 0.7	1.0 ± 0.7
	Milk, yoghurt and cheese (serves/day)	1.8 ± 1.1	1.4 ± 0.9	1.4 ± 0.9
	Non-animal protein foods (legumes, nuts, tofu) (serves/day)	0.3 ± 0.4	0.3 ± 0.5	0.3 ± 0.5
	UP EDNP foods (serves/day)	2.8 ± 1.8	2.7 ± 1.4	2.7 ± 1.4
	UP beverages (SSBs and alcohol) (serves/day)	1.3 ± 1.4	1.0 ± 1.0	1.0 ± 1.0
	Individually packaged EDNP foods and beverages (number of items)	2.1 ± 2.0	1.9 ± 1.4	1.9 ± 1.4
	Individually packaged healthy foods and beverages (number of items)	1.5 ± 1.2	1.6 ± 1.2	1.6 ± 1.2
	Food (plate) waste (% of total food)	11.1 ± 15.3	20 ± 15.1	20 ± 15.1

* A higher HSDI score indicates closer alignment to a healthy and sustainable diet; [26] ^a^ International Physical Activity Questionnaire; ^b^ Socio-Economic Indexes for Areas [26]; ^c^ Dietary health consciousness was determined by asking “Which of the following best describes how you feel about your diet?”; ^d^ Serve size according to the Australian Guide to Healthy Eating [6].

**Table 3:** Differences between total HSDI score * tertiles at baseline, using One-way ANOVA (continuous variables) and Chi-Squared test (categorical variables) (*n* = 246).

Variable	Description	Lowest Tertile (HSDI Score 20–38) *n* = 88	Middle Tertile (HSDI Score 39–46) *n* = 77	Highest Tertile (HSDI Score 47–69) *n* = 81	
		Mean ± SD	Mean ± SD	Mean ± SD	*p*-Value
Age	Years	24.4 ± 3	24.1 ± 3.6	24.4 ± 3.6	0.830
BMI	kg/m^2^	25.1 ± 5.9	23.6 ± 4.1	24.1 ± 5.8	0.162
		n (%)	n (%)	n (%)	*p*-Value
Sex	Men	29 (33.0)	27 (35.1)	29 (35.8)	0.921
	Women	59 (67.0)	50 (64.9)	52 (64.2)	
BMI	Healthy weight and below (<25 kg/m^2^)	54 (61.4)	57 (74.0)	56 (69.1)	0.418
	Overweight (25–29.9 kg/m^2^)	21 (23.9)	14 (18.2)	14 (17.3)	
	Obese (≥30 kg/m^2^)	13 (14.8)	6 (7.8)	11 (13.6)	
Vitamin supplement use	Yes	25 (28.4)	25 (32.5)	42 (51.9)	**<0.005**
	No	63 (71.6)	52 (67.5)	39 (48.1)	
Smoking status	Never smoked	54 (61.4)	55 (71.4)	60 (74.1)	0.212
	Previous smoker	26 (29.5)	20 (26.0)	18 (22.2)	
	Current smoker	8 (9.1)	2 (2.6)	3 (3.7)	
IPAQ category ^a^	Low activity (<600 MET mins/week)	11 (13.6)	9 (12.7)	12 (15.4)	0.988
	Moderate activity (minimum 600 MET mins/week)	44 (54.3)	40 (56.3)	41 (52.6)	
	High activity (>3000 MET mins/week)	26 (32.1)	22 (31.0)	25 (32.1)	
Ethnicity	White	74 (84.1)	56 (72.7)	60 (74.1)	0.283
	Asian	12 (13.6)	15 (19.5)	14 (17.3)	
	Other	2 (2.3)	6 (7.8)	15 (6.1)	
Education	Year 10, 11 or 12	33 (37.5)	27 (35.1)	28 (34.6)	0.947
	Trade or diploma	22 (25.0)	20 (26.0)	18 (22.2)	
	University degree or higher	33 (37.5)	30 (39.0)	35 (43.2)	
SEIFA ^b^	1–2	2 (2.3)	3 (3.9)	2 (2.5)	0.487
	3–4	2 (2.3)	5 (6.5)	7 (8.6)	
	5–6	27 (30.7)	18 (23.4)	15 (18.5)	
	7–8	15 (17.0)	15 (19.5)	20 (24.7)	
	9–10	42 (47.7)	36 (46.8)	37 (45.7)	
Dietary health consciousness ^c^	Pay a lot of attention to the health aspects of food	4 (4.7)	8 (10.4)	28 (34.6)	**<0.0005**
	Take a bit of notice to the health aspects of food	55 (64.0)	48 (62.3)	44 (54.3)	
	Don’t think much or don’t think at all	27 (31.4)	21 (27.3)	9 (11.1)	
**Individual HSDI item scores**		**Mean ± SD**	**Mean ± SD**	**Mean ± SD**	***p*** **-Value**
HSDI item scores of 0–10	Fruit	2.8 ± 2.6	4.7 ± 3.0	6.4 ± 3.1	**<0.0005**
	Vegetables	3.1 ± 1.7	3.8 ± 2.0	5.1 ± 2.2	**<0.0005**
	Non-animal protein foods (legumes, nuts, seeds, tofu)	1.1 ± 1.4	1.5 ± 1.5	2.6 ± 2.6	**<0.0005**
	Ultra-processed EDNP foods	0.8 ± 1.5	1.9 ± 2.2	3.6 ± 2.5	**<0.0005**
	Ultra-processed EDNP beverages (SSBs and alcohol)	2.8 ± 2.7	5.4 ± 3.5	7.8 ± 2.5	**<0.0005**
	Individually packaged healthy foods and beverages	4.5 ± 2.3	5.3 ± 2.8	6.4 ± 2.7	**<0.0005**
**Individual HSDI item scores**		**Mean ± SD**	**Mean ± SD**	**Mean ± SD**	***p*** **-value**
HSDI item scores of 0–5	Seasonal fruits and vegetables	3.0 ± 1.1	3.0 ± 1.1	3.1 ± 0.9	0.699
	Ruminant meat and pigs	3.6 ± 1.6	3.8 ± 1.6	3.8 ± 1.6	0.550
	Poultry, fish and eggs	3.9 ± 1.3	4.0 ± 1.2	4.4 ± 0.8	**<0.05**
	Milk, yoghurt and cheese	2.9 ± 1.1	2.9 ± 1.1	3.1 ± 1.1	0.644
	Individually packaged EDNP foods and beverages	1.0 ± 1.4	2.1 ± 1.7	3.4 ± 1.5	**<0.0005**
	Food (plate) waste	3.6 ± 1.4	3.9 ± 1.2	4.2 ± 1.2	**<0.05**

Statistically significant *p* values are in bold. * A higher HSDI score indicates closer alignment to a healthy and sustainable diet; ^a^ International Physical Activity Questionnaire; ^b^ Socio-Economic Indexes for Areas [26]; ^c^ Dietary health consciousness was determined by asking, “Which of the following best describes how you feel about your diet?”.

**Table 4:** Relationship between components of the HSDI at baseline, assessed using Spearman’s correlation coefficient (*n* = 246).

Spearman’s rho	Fruit										
Vegetables	0.307 **(*p* < 0.001)**	Vegetables									
Seasonal fruits & vegetables	–0.09 (*p* = 0.162)	–0.061 (*p* = 0.342)	Seasonal fruits & vegetables								
Ruminant meat & pigs	–0.093 (*p* = 0.146)	–0.225 **(*p* < 0.001)**	–0.044 (*p* = 0.488)	Ruminant meat & pigs							
Poultry, fish & eggs	–0.014 (*p* = 0.832)	0.027 (*p* = 0.673)	–0.023 (*p* = 0.722)	0.045 (*p* = 0.481)	Poultry, fish & eggs						
Milk, yoghurt & cheese	0.132 **(*p* < 0.05)**	0.136 **(*p* < 0.05)**	–0.108 (*p* = 0.090)	–0.062 (*p* = 0.332)	–0.011 (*p* = 0.868)	Milk, yoghurt & cheese					
Non-animal protein foods	0.258 **(*p* < 0.001)**	0.242 **(*p* < 0.001)**	–0.125 **(*p* < 0.05)**	–0.118 (*p* = 0.064)	–0.063 (*p* = 0.328)	0.138 **(*p* < 0.05)**	Non-animal protein foods				
Ultra-processed EDNP foods	0.082 (*p* = 0.200)	0.140 **(*p* < 0.05)**	0.04 (*p* = 0.536)	0.038 (*p* = 0.551)	0.076 (*p* = 0.234)	–0.086 (*p* = 0.180)	0.066 (*p* = 0.304)	Ultra-processed EDNP foods			
Ultra-processed EDNP drinks	0.111 (*p* = 0.083)	0.017 (*p* = 0.796)	–0.04 (*p* = 0.529)	–0.026 (*p* = 0.689)	0.083 (*p* = 0.193)	–0.106 (*p* = 0.098)	0.121 (*p*=0.058)	0.231 **(*p* < 0.001)**	Ultra-processed EDNP drinks		
Individually packaged EDNP items	0.080 (*p* = 0.209)	0.217 **(*p* < 0.001)**	–0.004 (*p* = 0.956)	0.044 (*p* = 0.494)	0.068 (*p* = 0.292)	–0.116 (*p* = 0.070)	0.07 (*p*=0.277)	0.322 **(*p* < 0.001)**	0.432 **(*p* < 0.001)**	Individually packaged EDNP items	
Individually packaged healthy items	–0.086 (*p* = 0.178)	–0.037 (*p* = 0.563)	0.116 (*p* = 0.070)	0.024 (*p* = 0.714)	0.147 **(*p* < 0.05)**	–0.233 **(*p* < 0.001)**	–0.071 (*p*=0.268)	0.107 (*p* = 0.094)	0.065 (*p* = 0.310)	0.132 **(*p* < 0.05)**	Individually packaged healthy items
Food (plate) waste	0.05 (*p* = 0.431)	0.099 (*p* = 0.120)	0.016 (*p* = 0.808)	–0.147 **(*p* < 0.05)**	0.026 (*p* = 0.682)	0.141 **(*p* < 0.05)**	–0.08 (*p*=0.212)	0.038 (*p* = 0.555)	0.034 (*p* = 0.599)	0.097 (*p* = 0.128)	0.019 (*p* = 0.765)

Statistically significant *p* values are in bold.

**Table 5:** Association between variables and the likelihood of being in the lowest tertile of HSDI scores * at baseline: Univariate; after adjusting for Age, Sex, BMI, and; Multivariable (*n* = 246).

Variable	Description	Univariate OR (95% CI) *p*-Value	After Adjusting for Age, Sex, BMI OR (95% CI) *p*-Value	Multivariable OR (95% CI) *p*-Value
Age	Years	1.017 (0.941, 1.098) *p* = 0.673		-
Sex	Women	1		-
	Men	0.895 (0.516, 1.554) *p* = 0.694		-
BMI	kg/m^2^	1.045 (0.995, 1.097) *p* = 0.076		-
Vitamin Supplements	Yes	1	1	-
	No	1.855 (1.059, 3.250) ***p* < 0.05**	1.810 (1.021, 3.209) ***p* < 0.05**	-
Smoking	Never smoked	1	1	-
	Previous smoker	1.457 (0.804, 2.640) *p* = 0.215	1.395 (0.757, 2.571) *p* = 0.286	-
	Current smoker	3.407 (1.065, 10.904) ***p* < 0.05**	3.284 (0.983, 10.964) *p* = 0.053	-
Ethnicity	White	1	1	-
	Asian	0.649 (0.312, 1.350) *p* = 0.247	0.743 (0.348, 1.585) *p* = 0.442	-
	Other	0.241 (0.053, 1.099) *p* = 0.066	0.201 (0.042, 0.971) ***p* < 0.05**	-
Education	Year 10,11 or 12	1.182 (0.648, 2.157) *p* = 0.586	1.330 (0.660, 2.678) *p* = 0.425	-
	Trade or diploma	1.140 (0.583, 2.232) *p* = 0.702	1.073 (0.529, 2.176) *p* = 0.846	-
	University degree or higher	1	1	-
SEIFA ^a^	1–2	0.695 (0.129, 3.742) *p* = 0.672	0.736 (0.135, 4.018) *p* = 0.723	-
	3–4	0.290 (0.062, 1.357) *p* = 0.116	0.256 (0.054, 1.225) *p* = 0.088	-
	5–6	1.422 (0.754, 2.683) *p* = 0.277	1.327 (0.695, 2.537) *p* = 0.391	-
	7–8	0.745 (0.365, 1.521) *p* = 0.419	0.695 (0.333, 1.447) *p* = 0.330	-
	9–10	1	1	-
IPAQ category ^b^	Low activity (<600 MET mins/week)	0.947 (0.396, 2.266) *p* = 0.902	0.906 (0.370, 2.220) *p* = 0.829	-
	Moderate activity (minimum 600 MET mins/week)	0.982 (0.537, 1.796) *p* = 0.953	1.011 (0.545, 1.876) *p* = 0.972	-
	High activity (>3000 MET mins/week)	1	1	-
Dietary health consciousness ^c^	Pay a lot of attention to the health aspects of food	1	1	1
	Take a bit of notice to the health aspects of food	5.380 (1.817, 15.934) ***p* < 0.005**	5.250 (1.765, 15.619) ***p* < 0.005**	5.276 (1.775, 15.681) ***p* < 0.005**
	Don’t think much or don’t think at all	8.100 (2.548, 25.747) ***p* < 0.0001**	8.152 (2.530, 26.272) ***p <* 0.0001**	8.308 (2.572, 26.836) ***p* < 0.0001**

Statistically significant *p* values are in bold. * A higher HSDI score indicates closer alignment to a healthy and sustainable diet; ^a^ Socio-Economic Indexes for Areas [26]; ^b^ International Physical Activity Questionnaire; ^c^ Dietary health consciousness was determined by asking “Which of the following best describes how you feel about your diet?”.

**Table 6:** Paired-sample *t*-test to assess the test–re-test reliability of the HSDI between data collected baseline and six months. Presented as HSDI scores * on participants who completed the study (*n* = 220).

	Description of Individual HSDI Item Scores	Baseline Visit Mean Score ± SD	6-Month Visit Mean Score ± SD	Mean Difference	*p*-Value
Items with score 0–10 points	Fruit	4.7 ± 3.3	4.1 ± 3.3	−0.6	**<0.05**
	Vegetables	3.9 ± 2.2	4.5 ± 2.4	0.5	**<0.001**
	Non-animal protein foods (legumes, nuts, seeds, tofu)	1.8 ± 2.1	1.8 ± 2.1	−0.0	0.821
	Ultra-processed EDNP foods	2.1 ± 2.4	3.0 ± 2.9	0.9	**<0.0005**
	Ultra-processed EDNP beverages (SSBs and alcohol)	5.2 ± 3.5	6.0 ± 3.5	0.8	**<0.005**
	Individually packaged healthy foods and beverages	5.4 ± 2.7	6.0 ± 2.8	0.6	**<0.005**
Items with score 0–5 points	Seasonal fruits and vegetables	3.0 ± 1.1	3.6 ± 1.2	0.7	**<0.0005**
	Ruminant meat and pigs	3.7 ± 1.6	4.1 ± 1.3	0.3	**<0.01**
	Poultry, fish and eggs	4.1 ± 1.1	4.3 ± 0.9	0.1	0.129
	Milk, yoghurt and cheese	3.0 ± 1.1	2.6 ± 1.1	−0.4	**<0.0005**
	Individually packaged EDNP foods and beverages	2.1 ± 1.7	2.9 ± 1.8	0.8	**<0.0005**
	Food (plate) waste	3.8 ± 1.3	4.2 ± 1.2	0.3	**<0.005**
Total score	Out of 90	42.8 ± 9.4	46.9 ± 10.2	4.1	<0.0005

Statistically significant *p* values are in bold. * A higher HSDI score indicates closer alignment to a healthy and sustainable diet.

## Data Availability

Data will be provided upon request.
